# Lower Plasma Elabela Levels in Hypertensive Patients With Heart Failure Predict the Occurrence of Major Adverse Cardiac Events: A Preliminary Study

**DOI:** 10.3389/fcvm.2021.638468

**Published:** 2021-03-02

**Authors:** Zheng Ma, Lei Zhao, Sara Martin, Yeping Zhang, Ying Dong, Jiu-Chang Zhong, Xin-Chun Yang

**Affiliations:** ^1^Heart Center and Beijing Key Laboratory of Hypertension, Beijing Chaoyang Hospital, Capital Medical University, Beijing, China; ^2^Santa Rosa Family Medicine Residency, Santa Rosa, CA, United States

**Keywords:** heart failure, hypertension, Elabela, prognosis factor, major adverse cardiac events

## Abstract

**Background:** Elabela, a novel cardiac developmental peptide, has been shown to improve heart dysfunction. However, the roles and correlation of Elabela in predicting adverse cardiac events in hypertensive patients with heart failure (HF) remain largely unclear.

**Objective:** To measure plasma levels of Elabela in hypertensive patients with HF and evaluate its prognostic value.

**Methods:** A single-site, cohort, prospective, observational study was investigated with all subjects, including control subjects and hypertensive patients with or without HF, whom were recruited in Beijing Chaoyang Hospital Affiliated to Capital Medical University form October 2018 to July 2019. The subjects among different groups were matched based on age and sex. The clinical characteristics were collected, and plasma Elabela levels were detected in all subjects. The hypertensive patients with HF were followed up for 180 days, and the major adverse cardiac events (MACE) were recorded. The Cox regression was used to explore the correlation between Elabela level and MACE in hypertensive patients with or without HF. The receiver operating characteristic curves were used to access the predictive power of plasma Elabela level.

**Results:** A total of 308 subjects, including 40 control subjects, 134 hypertensive patients without HF, and 134 hypertensive patients with HF were enrolled in this study. Plasma levels of Elabela were lower in hypertensive patients compared with control subjects [4.9 (2.8, 6.7) vs. 11.8 (9.8, 14.0) ng/ml, *P* < 0.001]. Furthermore, HF patients with preserved ejection fraction had a higher plasma Elabela level than those with impaired left ventricular systolic function (heart failure with mid-range ejection fraction and heart failure with reduced ejection fraction). The hypertensive patients with HF and higher plasma Elabela levels had a better readmission-free and MACE-free survival than those with lower plasma Elabela levels in survival analysis. The Cox regression analysis revealed that plasma Elabela levels were negatively associated with MACE (HR 0.75, 95% CI 0.61–0.99, *P* = 0.048) in hypertensive patients with HF.

**Conclusion:** Plasma Elabela levels were decreased in hypertensive patients with left ventricular systolic dysfunction. Thus, Elabela may be potentially used as a novel predictor for MACE in hypertensive patients with HF.

**Graphical Abstract:**
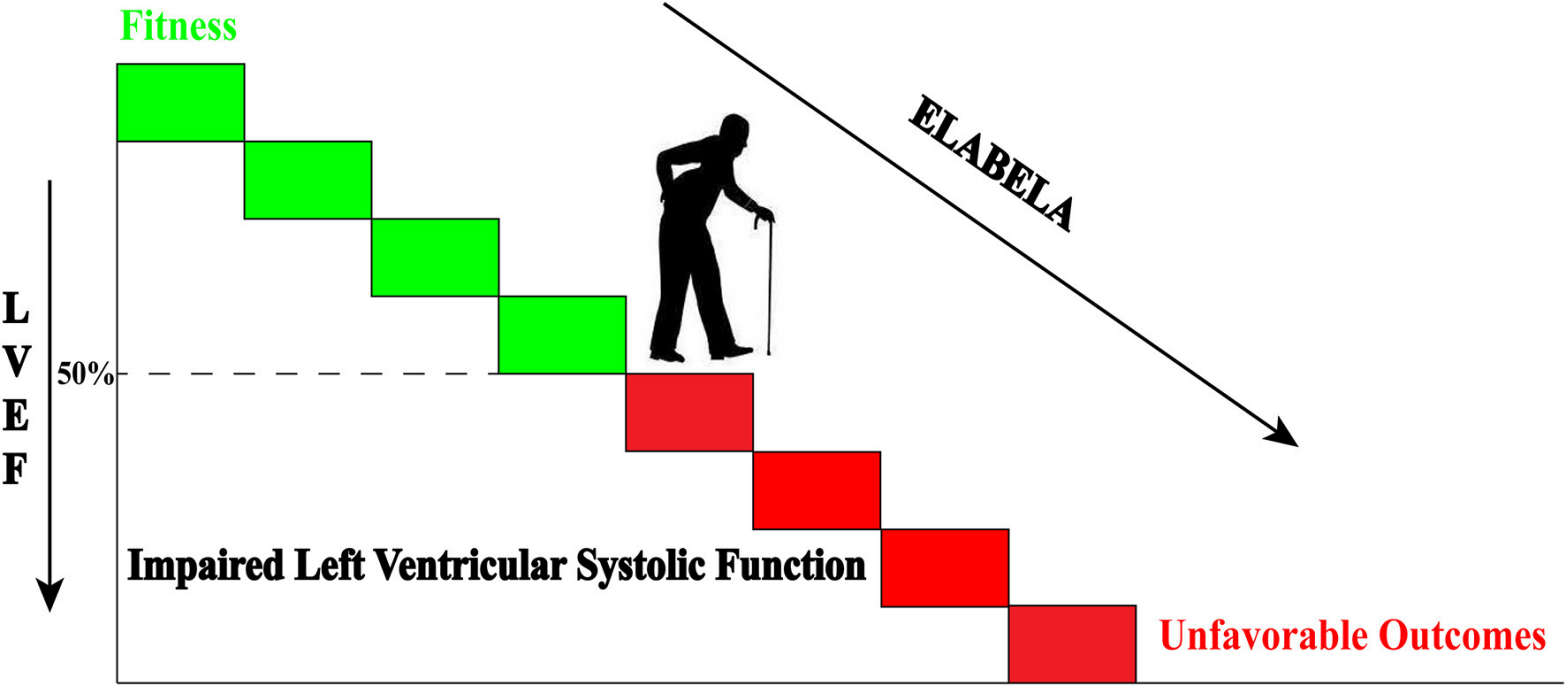
Declined plasma Elabela levels are associated with impaired left ventricular systolic function of hypertensive patients. The declined plasma Elabela levels predicated the unfavorable outcomes in hypertensive patients with heart failure during a 180-day follow-up.

## Introduction

Congestive heart failure (HF), which is often accompanied by multiple comorbidities, is a leading cause of mortality and morbidity worldwide (1). Hypertension, as one of the possible causes of heart failure, has a rapidly increasing incidence with the aging population. The optimized comprehensive management has greatly improved the outcomes of hypertensive patients, except those with HF. Early identification and effective risk stratification are crucial for the management of these patients (2, 3). BNP has become a widely-used biomarker and valuable adverse events predictor for patients with HF (4). However, its low specificity limits its predictive power in a clinical application (5).

Elabela (also called Toddler or Apela) was identified as a novel endogenous ligand of the APJ receptor that had an important role in cardiac development (6). Further study found that Elabela also exerts the important biological effects (anti-hypertension, positive inotropic action, diuresis, anti-remodeling, antifibrotic action, as well as cardiorenal protection) in adult animals through Elabela/APJ signaling (7, 8). Clinical studies suggested that patients with hypertension had lower plasma Elabela levels than a healthy control group (9), and plasma Elabela levels were negatively associated with the extent of albuminuria in patients with type 2 diabetes (10).

Recent preclinical studies further confirmed that Elabela/APJ axis could prevent pressure overload HF and angiotensin II-induced cardiac damage through depressing ACE and FoxM1 expression and activating ERK1/2 pathway (11). The Elabela also improved hemodynamic parameters, including increased E-wave velocity and left ventricular end-diastolic volume (12). These results indicated that Elabela might take part in the prevention of HF. The correlation between Elabela and patients with hypertension or albuminuria (that are both independent risk factors for HF) suggested that Elabela may be an important biomarker for HF (13–15). So far, no studies have investigated plasma Elabela level and its prognostic value in patients with HF. Thus, in the present study, we measured the plasma Elabela levels and investigated the association between plasma Elabela and the outcomes in hypertensive patients with HF.

## Materials and Methods

### Study Population

This was a single-site, cohort, prospective, and observational study. All subjects were recruited in the Heart Centre of Beijing Chaoyang Hospital, Capital Medical University, between October 2018 and July 2019. Hypertensive patients with or without HF were consecutively recruited into the HF group and non-HF group, respectively. The control subjects without cardiovascular diseases from our Health Examination Center were consecutively enrolled in the healthy control group during the same period. The subjects from different groups were matched 1:1 based on the same-sex with a maximum age difference of 5 years. The exclusion criteria were: (1) congenital heart disease; (2) cardiomyopathy; (3) severe renal dysfunction with estimated glomerular filtration rate (eGFR) ≤30 ml/min/1.73 m^2^ at baseline; (4) tumor; (5) severe infection, autoimmune disease, and mental disorder; (6) any other non-cardiovascular diseases which lead the life expectancy of fewer than 6 months; (7) acute exacerbation of chronic bronchitis and exacerbated asthma. Written informed consent and clinical characteristics were obtained from all subjects at the time of enrollment. All the laboratory assessments, except plasma Elabela levels, were conducted in the clinical laboratory center according to the standard protocols. The eGFR were estimated by using MDRD Study China equation [eGFR = 175×(serum creatinine mg/dl)^−1.234^×age^−0.179^×0.79 (if female)] (16).

All echocardiogram measurements were obtained by two experienced attending doctors. All of the HF patients received the optimized treatment as outlined in the 2016 ESC Guidelines (4). The flow diagram of the study (from enrollment to follow-up) was shown in Supplementary Figure 1.

### Diagnostic Criteria

Criteria for hypertension diagnosis were: (1) systolic blood pressure (SBP) ≥140 mmHg and/or diastolic blood pressure (DBP) ≥90 mmHg in the office or clinic following repeated examination, (2) SBP ≥135 mmHg and/or DBP ≥85 mmHg at home, (3) 24-h average SBP ≥130 mmHg and/or DBP ≥80 mmHg, day time average SBP ≥135 mmHg and/or DBP ≥85 mmHg, or night time average SBP ≥120 mmHg and/or DBP ≥70 mmHg in ambulatory blood pressure monitoring (17). Criteria for diagnosis and classification of HF were based on the 2016 ESC Guidelines for the diagnosis and treatment of HF (4). Briefly, the typical signs (dyspnea), symptoms (crackles on lung auscultation), elevated brain natriuretic peptide (BNP) levels, X-ray examination (signs of pulmonary congestion and enlarged heart shadow), and ultrasound cardiogram report (impaired left ventricular diastolic and or systolic function) were all considered when the HF diagnosis was made. Heart failure with reduced ejection fraction (HFrEF) was defined as EF ≤40% (4); heart failure with preserved EF (HFpEF) as EF ≥50% (4); heart failure with mid-range EF (HFmrEF) as EF between 41 and 49% (4). The optimized treatment of HF was received but was not limited to the usage of diuretics, renin-angiotensin-aldosterone system antagonist, and beta-blockers (4).

### Elabela Enzyme Immunoassay

All the blood samples were collected from a peripheral vein. Upon collection, venous blood samples were immediately processed with a centrifuge at 4°C and 3,000 rpm for 10 min. Plasma samples were then stored at −80°C until use. The commercialized human Elabela Elisa Kit (S-1508, Peninsula Laboratories International, Inc. USA) was used to measure plasma Elabela level with the test range: 0–100 ng/ml and average IC50: 2 ng/ml. The operation procedures followed the instructions of Elisa Kit. As the instructions suggested, the samples were appropriately extracted.

### Follow-Up and Endpoints

The primary follow-up endpoint was the occurrence of major cardiac adverse events (MACE), including all-cause mortality and HF readmission. The length of hospital stay was used as the secondary endpoint. All 134 hypertensive patients with HF were divided into two groups (high-level group and low-level group) by the median of plasma Elabela level and then were followed up for 180 days (from November 2018). Telephone follow-up was conducted at a fixed time every month (Supplementary Figure 2).

### Statistical Analysis

Continuous data were presented as mean ± standard deviation (SD) or median and interquartile range (IQR), and categorical data as number and percentage. Student's *t*-test was used for normally distributed variables when comparing continuous variables between two groups, and Mann–Whitney *U*-tests for non-normally distributed variables. In a comparison of the continuous variable among more than two groups, one-way analysis of variance was used for normally distributed variables, and the Kruskal-Wallis test was used for non-normally distributed variables. The Student Newman Keuls test was employed in pairwise comparison among three groups, and the adjusted *p*-value was provided. Fisher's exact test was used for categorical variables. BNP value was seriously skewed, and the logarithmic transformation was used for data conversion. Spearman correlation analyses were used to assess the relationship between plasma Elabela levels and study variables, including age, sex, BMI, Log_10_ BNP, blood lipid, renal function, and echocardiographic parameters. Kaplan–Meier survival curves with a log-rank test analyzed MACE, HF readmission, and survival. Cox regression was invited to explore the predictors of MACE in hypertensive patients with HF. Only variables with *P* < 0.10 in univariate analysis were included in the multivariable model. All continuous variables which entered the Cox regression met the linearity assumption. To analyze the predictive power of selected predictors, receiver operating characteristic curves (ROC) were calculated, and the area under the curve (AUC) was determined. The hazard ratio (HR) and 95% confidence intervals (CI) were reported. The MACE predictive cut-off point was selected according to the Youden index. All tests were 2-sided, and statistical significance was set at a value of *P* < 0.05. The statistical analysis was performed using the SPSS software version 23 (IBM Corporation, Armonk, NY).

## Results

### The Baseline Characteristics of Hypertensive Patients

The baseline characteristics of 268 hypertensive patients (134 patients with HF and 134 age sex-matched patients without) were shown in Table 1. Data from laboratory examinations revealed that plasma BNP levels, serum creatinine levels, hemoglobin A1C levels, and high-sensitivity C-reactive protein (hs-CRP) levels were all higher in the HF group compared to the non-HF group (*P* < 0.05). In contrast, the high-density lipoprotein cholesterol (HDL-c) levels were significantly lower in the HF group compared to the non-HF group. Significant differences were also observed between the HF and non-HF group in echocardiographic parameters, including left atrial diameter, left ventricular end-diastolic diameter, left ventricular end-systolic diameter, pulmonary arterial pressure (PASP), and left ventricular ejection fraction (LVEF).

**Table 1:** Baseline characteristics and laboratory data of hypertensive patients.

	**Total**	**Non-HF group**	**HF group**	***P***
	**(*n* = 268)**	**(*n* = 134)**	**(*n* = 134)**	
Age, years	67.8 ± 10.8	67.8 ± 10.5	68.8 ± 11.1	0.426
Male sex	173 (64.6%)	87/134 (64.9%)	86/134 (64.2%)	0.898
BMI, kg/m^2^	25.5 ± 3.3	25.7 ± 3.2	25.3 ± 3.4	0.295
**Comorbidities**				
Coronary heart disease	185/268 (69%)	89/134(66.4%)	96/134 (71.6%)	0.355
Atrial fibrillation	118/268 (44.0%)	57/134 (42.5%)	61/134 (45.5%)	0.377
Diabetes Mellitus	120/268 (44.8%)	56/134 (41.8%)	64/134 (47.8%)	0.326
Chronic renal failure	35/268 (13.1%)	7/134 (5.2%)	28/134 (20.9%)	<0.001
Hyperlipidemia	173/268 (64.6%)	87/134 (64.9%)	86/134 (64.2%)	0.898
**Laboratory data**				
BNP level, pg/ml	151.0 (55.3, 560.5)	59.5 (26.7, 132.5)	506.0 (194.7, 1438.7)	<0.001
Creatine level, umol/l	72.4 (62.8, 82.1)	70.7 (61.0, 80.5)	76.4 (64.9, 114.5)	<0.001
eGFR, ml/(min·1.73 m^2^)	84.4 ± 30.6	91.7 ± 32.3	77.2 ± 26.8	0.027
Hemoglobin A1C, %	6.4 ± 1.0	6.3 ± 1.0	6.6 ± 1.1	0.040
Triglyceride, mmol/l	1.4 ± 0.8	1.5 ± 0.9	1.3 ± 0.7	0.140
LDL-c, mmol/l	2.3 ± 0.9	2.3 ± 0.8	2.3 ± 0.9	0.606
HDL-c, mmol/l	1.0 ± 0.3	1.1 ± 0.3	0.9 ± 0.3	0.002
Total cholesterol, mmol/l	4.1 ± 1.1	4.2 ± 1.0	4.0 ± 1.2	0.107
Hs-CRP, mg/l	2.7 (1.1, 9.0)	1.6 (0.8, 3.2)	6.3 (2.4, 14.0)	<0.001
Troponin I, ng/ml	0.02 (0.00, 0.10)	0.01 (0.00, 0.02)	0.05 (0.02, 0.18)	<0.001
Elabela, ng/ml	4.4 (2.3, 6.2)	4.7 (3.0, 7.4)	3.9 (1.9, 5.4)	<0.001
**Echcardiography**				
LAD, mm	42.3 ± 7.1	39.9 ± 6.7	44.6 ± 7.4	<0.001
LVEDd, mm	51.4 ± 4.5	47.0 ± 4.2	55.1 ± 4.8	<0.001
LVEDs, mm	35.9 ± 5.6	29.7 ± 5.0	41.3 ± 6.2	<0.001
PASP, mmHg	27.1 (24.5, 29.4)	25.9 (24.2, 27.5)	28.9 (25.4, 48.3)	<0.001
LVEF, %	60.0 (42.0, 66.0)	65.0 (61.0,69.0)	44.0 (37.8, 58.0)	<0.001
**Nyha function class**				
Class II	44/268 (16.4%)	NA	44/134 (32.8%)	NA
Class III	44/268 (16.4%)	NA	44/134 (32.8%)	NA
Class IV	46/268 (17.2%)	NA	46/134 (34.3%)	NA

*eGFR, estimated glomerular filtration rate; LDL-c, low-density lipoprotein cholesterol; HDL-c, high-density lipoprotein cholesterol; hs-CRP, high-sensitivity C-reactive protein; LAD, left atrial diameter; LVEDd, left ventricular end-diastolic dimension; LVEDs, left ventricular end-systolic diameter; PASP, pulmonary arterial pressure; LVEF, left ventricular ejection fraction; NA, not available*.

### Plasma Elabela Levels in Hypertensive Patients With HF and Without HF

Due to the differences in age among control subjects and hypertensive patients, the Elabela plasma levels in 40 control subjects (45.0% female, mean age 56.6 ± 6.0 years) and 40 age and sex-matched hypertensive patients with or without HF from the 268 hypertensive patients (45.0% female, mean age 57.5 ± 5.6 years) were compared. Plasma Elabela levels were significantly lower in hypertensive patients with or without HF compared to control subjects [4.9 (2.8, 6.7) vs. 11.8 (9.8, 14.0) ng/ml, *P* < 0.001]. Moreover, plasma Elabela levels were significantly lower in hypertensive patients with HF when compared with control subjects [3.0 (1.9, 4.9) vs. 11.8 (9.8, 14.0) ng/ml, *P* < 0.001] (Supplementary Figure 3).

**Figure 1:**
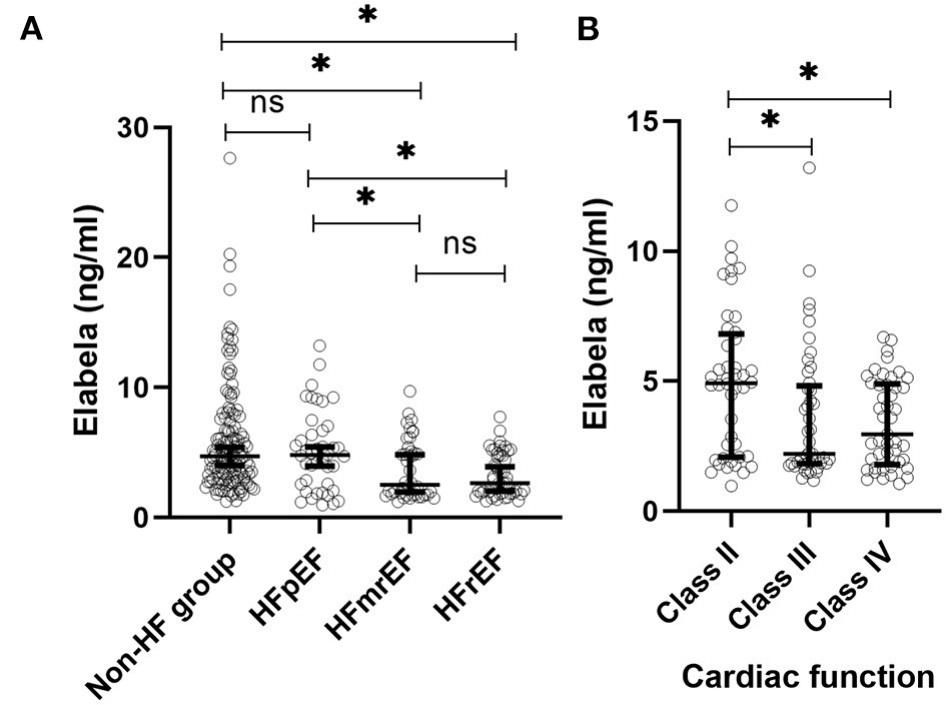
Plasma Elabela levels in hypertensive patients with different types of HF. **(A)** The mean plasma Elabela level of the non-HF group were similar with that of HFpEF group. Plasma Elabela levels of patients with HFrEF were similar with that of HFmrEF. Furthmore, patients without HF had a higher mean plasma level of Elabela compared with patients with HFrEF and HFmrEF separately [4.7 (3.0, 7.4) vs. 2.6 (1.9, 4.9) ng/ml, *P* = 0.01 and 4.7 (3.0, 7.4) vs. 2.7 (1.8, 5.4) ng/ml, *P* < 0.001 separately]. Importanly, patients with HFpEF had higher plasma levels of Elabela than patients with HFrEF and HFmrEF separately [4.8 (2.4, 6.8) vs. 2.6 (1.9, 4.9) ng/ml, *P* = 0.010 and 4.8 (2.4, 6.8) vs. 2.7 (1.8, 5.4) ng/ml, *P* = 0.037 separately]. **(B)** There were no difference of plasma Elabela levels between HF patients with NYHA class III and IV; Plasma Elabela levels were significantly higher in HF patients with NYHA class II than those with NYHA class III and class IV separately [4.9 (2.1, 6.8) vs. 2.2 (1.8, 4.8), *P* = 0.007 and 4.9 (2.1, 6.8) vs. 3.0 (1.8, 4.9) ng/ml, *P* = 0.011 separately]. ELA, Elabela; HFrEF, heart failure with reduced ejection fraction; HFmrEF, heart failure with middle-range fraction; HFpEF, heart failure with preserved ejection fraction; NYHA, New York Heart Association.

In 268 hypertensive patients, plasma Elabela levels were significantly lower in the HF group compared to the non-HF group [3.9 (1.9, 5.4) vs. 4.7 (3.0, 7.4) ng/ml, *P* < 0.001]. We further divided the 134 patients with HF into HFrEF (50/134), HFmrEF (42/134), and HFpEF (42/134) groups according to LVEF. The mean plasma Elabela level of the Non-HF group and HFpEF group were similar [4.7 (3.0, 7.4) vs. 4.8 (2.4, 6.8) ng/ml, *P* = 0.999]. HFpEF group, like non-HF group, had higher plasma levels of Elabela than HFrEF and HFmrEF group [4.8 (2.4, 6.8) vs. 2.6 (1.9, 4.9) ng/ml, *P* = 0.010 and 4.8 (2.4, 6.8) vs. 2.7 (1.8, 5.4) ng/ml, *P* = 0.037 separately], while no significant differences were observed between HFrEF and HFmrEF groups. HF patients were further divided into another three subgroups (class II, III, and IV groups) according to the classification of NYHA. Intriguingly, plasma Elabela levels were significantly higher in the class II group of HF patients than in class III and class IV groups [4.9 (2.1, 6.8) vs. 2.2 (1.8, 4.8), *P* = 0.007 and 4.9 (2.1, 6.8) vs. 3.0 (1.8, 4.9) ng/ml, *P* = 0.011 separately] with no significant differences observed between class III and class IV groups (Figure 1).

### Correlation Between Elabela and Study Variables

We further analyzed the correlation between Elabela and study variables in all subjects (Supplementary Table 1). Edema(*r* = −0.23, *P* < 0.001), Third heart sound (*r* = −0.22, *P* < 0.001), Rales (*r* = −0.21, *P* < 0.001), Jugular venous distention(*r* = −0.20, *P* = 0.001), Log10 BNP (*r* = −0.20, *P* = 0.001), creatinine levels (*r* =-0.13, *P* = 0.029), troponin I levels (*r* = −0.19, *P* = 0.002), left atrial diameter (*r* = −0.14, *P* = 0.027), left ventricular end diastolic diameter (*r* = −0.34, *P* < 0.001), left ventricular end systolic diameter (*r* =-0.29, *P* < 0.001) and PASP (*r* = −0.27, *P* < 0.001) were negatively related to plasma Elabela levels, whereas eGFR (*r* = 0.13, *P* = 0.034) and LVEF (*r* = 0.23, *P* < 0.001) were positively correlated to plasma Elabela levels.

### Baseline Clinical Characteristics and Outcome of Patients With Different Levels of Elabela

**Figure 2:**
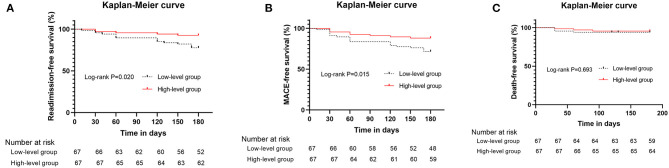
Kaplan–Meier curves for hypertensive patients with heart failure above and below the median values for plasma Elabela level. **(A)** Kaplan–Meier curves (heart failure readmission) for patients above and below the median values for plasma Elabela levels; **(B)** Kaplan–Meier curves (composite outcomes, MACE) for patients above and below the median values for plasma Elabela levels; **(C)** Kaplan–Meier curves (all-cause mortality) for patients above and below the median values for plasma Elabela levels.

According to the median of plasma Elabela level, all the hypertensive patients with HF were divided into two groups, the high-level group and the low-level group (Supplementary Table 2). Low-level group had more male patients (77.6 vs. 50.7%, *P* = 0.001), higher BNP levels [594.0 (342.0, 1917.0) vs. 367.0 (133.0, 1044.0) pg/ml, *P* = 0.032], lower total cholesterol levels (3.7 ± 1.2 vs. 4.2 ± 1.2 mmol/l, *P* = 0.049) and lower plasma Elabela levels [1.9 (1.6, 2.3) vs. 5.4 (4.8, 6.7) ng/ml, *P* < 0.001] than those in high-level group. Echocardiographic data indicated that the low-level group had larger atrial and ventricular chambers and worse left ventricular systolic function than the high-level group (*P* < 0.05). After the 180-day follow-up, 15 out of 67 patients (22.4%) from the low-level group were admitted for HF recurrence, while only 5 out of 67 patients (7.5%) from the high-level group were readmitted (*P* = 0.015). Although the all-cause mortality had no statistical difference between the two groups (6.0 vs. 4.5%, *P* = 0.698), the MACE rate in the low-level group was higher than those in the high-level group (28.4 vs. 11.9%, *P* = 0.018). The high-level group had better readmission-free and MACE-free survival (Figure 2). No significant difference was found in the median lengths of hospital stay between the two groups.

### Predictors of Baseline Characteristics for the Unfavorable Outcome of HF

To analyze the prognostic value of Elabela, we divided hypertensive patients with HF into a favorable outcome group (107 patients without MACE) and an unfavorable outcome group (27 patients with MACE). The baseline characteristics were shown in Supplementary Table 3. In univariate Cox proportional hazards analysis, log_10_ BNP levels [HR 5.05, 95% CI (2.28–11.17), *P* < 0.001], eGFR [HR 0.98, 95% CI (0.97–0.99), *P* = 0.006], plasma Elabela levels [HR 0.73, 95% CI (0.58–0.91), *P* = 0.006], classification of NYHA [HR 3.16, 95% CI (1.74–5.74), *P* < 0.001] and PASP [HR 1.03, 95% CI (1.00–1.05), *P* = 0.025] were closely associated with the occurrence of MACE. These factors were then incorporated into the multivariate analysis. Finally, plasma Elabela levels [HR 0.75, 95% CI (0.61–0.99), *P* = 0.048] and log_10_ BNP [HR 4.04, 95% CI (1.82–9.00), *P* = 0.001] were associated with the occurrence of MACE (Supplementary Table 4). ROC curve was used to assess the predictive value of plasma Elabela levels and BNP levels for the occurrence of MACE (Supplementary Figure 4). The AUC area of Elabela was 0.70 (95% CI 0.59–0.82), and the predictive cut-off point was 2.60 ng/ml (sensitivity 0.74, specificity 0.79). In contrast, the AUC area of log_10_ BNP was 0.76 (95% CI 0.67–0.85), and the predictive cut-off point was 2.58 ng/ml (sensitivity 0.93, specificity 0.50). Furthermore, the AUC area of the combination of Elabela and log_10_ BNP was 0.78 (95% CI 0.70–0.88). The predictive cut-off point of Elebala was 2.86 ng/ml, and log_10_ BNP was 2.58 (sensitivity 0.89, specificity 0.58).

## Discussion

To the best of our knowledge, this is the first study that investigated the predictive value of plasma Elabela levels in hypertensive patients with HF. Our study revealed that the declined plasma Elabela level was a promising predictor of HF readmission for HF patients. Moreover, we found that plasma Elabela levels were positively correlated with LVEF and negatively associated with the size of the left ventricle. These findings highlighted the need for conducting research on the biological action and mechanism of Elabela in the context of HF.

This study showed that plasma Elabela levels were significantly lower in hypertensive patients, especially in those with HF when compared with those in control subjects. A previous study reported lower plasma Elabela levels in patients with essential hypertension (9). The primary causes were recognized as the loss of hypotensive effect and endothelial protection from Elabela. So far, no study investigated plasma Elabela levels in other cardiovascular diseases such as coronary heart disease and atrial fibrillation. The differences in plasma Elabela levels among control subjects and hypertensive patients in this study revealed an underlying relationship between Elabela deficiency and cardiovascular diseases. In addition, due to the similar bioeffects with Apelin, it was indirectly implied that Elabela might be a protective factor preventing cardiovascular disease (18, 19). Apelin shared the same APJ receptor as Elabela and had been found to be significantly decreased in the plasma of HF patients (20–23). However, until now, plasma Elabela levels in HF patients had rarely been investigated. Our data showed that plasma Elabela levels in patients with HF were significantly depressed compared with those without HF. Elabela was essential for diverse biological processes and has important roles in regulating fluid homeostasis, myocardial contractility, vasodilation, angiogenesis, myocardial fibrosis, apoptosis and proliferation, thus, contributing to the prevention of HF (11, 12, 24, 25). Our findings also indirectly supported previous studies that showed lower concentrations of plasma Elabela in patients with hypertension and renal impairment (9, 10), which were independent risk factors for HF development.

We further analyzed plasma Elabela levels in different types of HF. The HF patients with HFrEF or HFmrEF had lower plasma Elabela levels than HF patients with HFpEF and patients without HF. Interestingly, neither plasma Elabela levels between the non-HF and HFpEF group, nor plasma Elabela levels between HFrEF and HFmrEF group showed notable differences. These results showed a close relationship between Elabela and impaired left ventricular systolic function. This may be attributed to the positive inotropic effect of Elabela that was previously demonstrated in animal research (12, 26). It has also been reported that Elabela limited the area of cardiac fibrosis and downregulated the expression of profibrotic genes (11). Therefore, HF development might be ascribed to the adverse left ventricular remodeling and the systolic dysfunction due to lower plasma Elabela levels. Consistent with the above results, plasma Elabela levels were lower in patients with worse NYHA classification. Pulmonary hypertension is an independent predictive factor for adverse events in patients with HF (27). Previous studies showed that Elabela expression in human pulmonary hypertension (PHT) lung was significantly reduced comparing with healthy lung (7). Consistent with the previous results, plasma Elabela levels was inversely associated with PASP in our study. The similar trends in both tissues and circulation indicated a strong relationship between Elabela and pulmonary arterial pressure. Based on this, the correlation between Elabela and PASP might become stronger in patients with HF and PHT who often has a worse prognosis.

The multiple bioeffects of Elabela have a vital role in the progression of HF. The signs of HF are important clues for HF diagnosis. The characteristic signs of HF include the Edema, third heart sound, rales, and jugular venous distention. Recently, it was reported that these signs had independent prognostic value even beyond symptoms and natriuretic peptides (28). We found that plasma Elabela levels were negatively correlated with these signs. These results indirectly indicated that plasma Elabela levels were associated with prognosis of HF. Chronic kidney disease and HF are closely related. They interact with each other and deteriorate patient's condition. Accordingly, kidney function is a well-established risk predictor in HF patients (29). In our study, the correlations between Elabela and eGFR and creatinine levels suggested that declined plasma Elabela levels might be associated with renal impairment. Evidence from the previous basic research and clinical study was in line with our findings. It was also reported that Elabela protected against podocyte injury in diabetic mice (30). In addition, declined plasma Elabela levels were associated with albuminuria in patients with type 2 diabetes (10). Given this evidence, declined plasma Elabela levels might increase the incidence of HF development via renal impairment and its dysfunction. Plasma Elabela levels had a positive correlation with HDL-c levels, which is a protective factor in cardiovascular diseases. This result revealed that Elabela might work as adipocytokines like Apelin taking part in metabolic regulation (31). Plasma levels of Elabela were much higher in patients with good cardiac function (NYHA class II) than those with poor cardiac function (NYHA class III and IV). These results showed a trend that patients with lower plasma Elabela level had an exacerbated cardiac dysfunction than those with higher Elabela plasma level. It is well-established that the worsen heart function is an independent risk factor for adverse events in patients with HF (32). The relationship between plasma Elabela level and heart function might be connected the declined plasma Elabela level to the adverse events in patients with HF. Notably, Elabela was also negatively related to both BNP and troponin I in our study. The relation between Elabela and BNP revealed that the anti-HF effects of Elabela might include the positive inotropic effect and the inhibition of cardiac remodeling. The negative relationship between Elabela and troponin I demonstrated the effect of Elabela on combating myocardial injury. The anti-inflammatory and antioxidant effects of Elabela might effectively prevent and limit myocardial injury. Hence, plasma levels of Elabela might be used as a new tool for the severity stratification of patients with HF in the future. Notably, further studies should also be conducted to investigate the interactions among Elabela, BNP, and troponin I.

It has been proved that BNP levels are associated with HF severity and mortality (33). Unfortunately, BNP has a poor predictive power on specificity (34). Our results suggest that Elabela might be a novel promising biomarker for HF severity. In our study, multivariate analysis revealed lower plasma Elabela levels as a useful predictor of a worse prognosis. Plasma Elabela levels were predictive for HF readmission and MACE. The predictive ability of Elabela might be attributed to its multiple protective effects, including antihypertensive effect, protection of renal function, inhibition of cardiac remodeling, suppression of inflammatory response, and impairment of myocardial injury (11, 12, 35). These results were in line with the previous conclusions advocating that BNP was an important predictor for adverse events in HF patients (36). Although the BNP had a significantly greater predictive sensitivity compared to Elabela, Elabela was superior to BNP in predictive specificity. Taking Elabela and BNP into consideration greatly improved physician's predictive ability for adverse events in HF patients. Importantly, our study revealed that the MACE was clearly driven by HF hospitalization, not by mortality. These results were due to the positive inotropic action of Elabela. Cardiac remodeling, an important impact factor on mortality, is a slow process (37). Thus, the short follow-up period might explain the lack of difference in mortality and a longer follow-up period is necessary for the certification of the long-term protective effect of Elabela for HF patients.

This study still had a few limitations. Firstly, the sample size was small and follow-up time was short. Secondly, control subjects in this study were younger than overall hypertensive patients with or without HF. So, we had to compare age and gender-matched patients. It still remained unknown whether there are differences between healthy volunteers and patients of advantage age. Thirdly, although the patients with and without HF had similar incidences of cardiovascular diseases in this study, confounders and interactions were inevitable.

## Conclusions

The present study demonstrated for the first time that plasma Elabela levels were declined in hypertensive patients with HF, especially in those with left ventricular systolic dysfunction. Plasma Elabela levels were associated with multiple risk factors for HF. Lower plasma Elabela level might be used as a promising predictor for MACE in hypertensive patients with HF.

## Data Availability Statement

The raw data supporting the conclusions of this article will be made available by the authors, without undue reservation.

## Ethics Statement

The studies involving human participants were reviewed and approved by Chaoyang Hospital. The patients/participants provided their written informed consent to participate in this study.

## Author Contributions

X-CY and J-CZ contributed to conception and design. ZM and LZ conducted the study and drafted the manuscript. YZ contributed to acquisition, analysis, and interpretation. YD contributed to analysis. SM critically revised the manuscript. All authors contributed to the article and approved the submitted version.

## Conflict of Interest

The authors declare that the research was conducted in the absence of any commercial or financial relationships that could be construed as a potential conflict of interest.

## Supplementary Material

Supplementary Figure 1 Flow diagram for study from enrollment to follow-up.

Supplementary Figure 2 Flow diagram for follow-up. Firstly, whether the subject is alive or not were confirmed. If the subject has died, the death details will be further investigated and death certificate will provide important information. Secondly, whether the subject is readmission or not were confirmed. If yes, we will further get the details information including the symptom, the diagnosis and medication records. Our team will clarify the reason of readmission. If no, we will ask the subject whether he/she had any symptoms associated with the deterioration of heart failure. If yes, we will assess the necessity of further outpatient visits and readmission.

Supplementary Figure 3 Plasma Elabela levels in control subjects and age-sex-matched hypertensive patients. **(A)** The comparison between control subjects and hypertensive patients; **(B)** The comparison among control subjects, hypertensive patients with and without HF.

Supplementary Figure 4 ROC curve of the prognostic value of plasma Elabela levels in predicting major adverse cardiac events in patients with HF.
