# Supplementary material for: Lower Plasma Elabela Levels in Hypertensive Patients With Heart Failure Predict the Occurrence of Major Adverse Cardiac Events: A Preliminary Study
Source: Front Cardiovasc Med. 2021 Mar 2;8:638468. doi: 10.3389/fcvm.2021.638468 (PMC7960768; doi:10.3389/fcvm.2021.638468)
Supplement: Supplementary file 1 [file Table_1.docx]

**Table S1.** Correlation between Elabela and Study Variables in All Subjects

|  | r | P-value |
| --- | --- | --- |
| Age | -0.11 | 0.061 |
| Male sex | 0.12 | 0.055 |
| BMI | 0.06 | 0.305 |
| Coronary heart disease | -0.06 | 0.370 |
| Atrial fibrillation | 0.08 | 0.219 |
| Diabetes Mellitus | -0.09 | 0.144 |
| Chronic renal failure | -0.09 | 0.124 |
| Hyperlipidemia | -0.06 | 0.367 |
| Edema | -0.23 | <0.001 |
| Third heart sound | -0.22 | <0.001 |
| Rales | -0.21 | <0.001 |
| Jugular venous distention | -0.20 | 0.001 |
| Log_10_ BNP | -0.20 | 0.001 |
| Creatine | -0.13 | 0.029 |
| eGFR | 0.13 | 0.034 |
| Hemoglobin A1C | -0.07 | 0.257 |
| Triglyceride | 0.08 | 0.194 |
| LDL-c | 0.07 | 0.262 |
| HDL-c | 0.11 | 0.076 |
| Total cholesterol | 0.06 | 0.128 |
| Troponin I | -0.19 | 0.002 |
| Hs-CRP | -0.13 | 0.066 |
| LAD | -0.14 | 0.027 |
| LVEDd | -0.34 | <0.001 |
| LVEDs | -0.29 | <0.001 |
| PASP | -0.27 | <0.001 |
| LVEF | 0.23 | <0.001 |

BNP, brain natriuretic peptide; eGFR, estimated glomerular filtration rate; LDL-c, low-density lipoprotein cholesterol; HDL-c, high-density lipoprotein cholesterol; hs-CRP, high-sensitivity C-reactive protein; LAD, left atrial diameter; LVEDd, left ventricular end-diastolic dimension; LVEDs, left ventricular end-systolic diameter; PASP, pulmonary arterial pressure; LVEF, left ventricular ejection fraction.
