# Supplementary material for: Lower Plasma Elabela Levels in Hypertensive Patients With Heart Failure Predict the Occurrence of Major Adverse Cardiac Events: A Preliminary Study
Source: Front Cardiovasc Med. 2021 Mar 2;8:638468. doi: 10.3389/fcvm.2021.638468 (PMC7960768; doi:10.3389/fcvm.2021.638468)
Supplement: Supplementary file 2 [file Table_2.docx]

**Supplementary Table 2. The Demographic and Baseline Characteristics of the HF Patients with** **Low level and High Level of Elabela**

|  | Low-level group  (n=67) | High-level group  (n=67) | P-value |
| --- | --- | --- | --- |
| **Age, years** | 67.7±12.6 | 70.0±9.4 | 0.232 |
| **Male sex** | 52/67 (77.6%) | 34/67 (50.7%) | 0.001 |
| **BMI, kg/m^2^** | 25.0±3.3 | 25.6±3.6 | 0.358 |
| **Comorbidities** |  |  |  |
| Coronary heart disease | 46/67 (68.7%) | 48/67 (71.6%) | 0.706 |
| Atrial fibrillation | 30/67 (44.8%) | 27/67 (40.3%) | 0.600 |
| Diabetes Mellitus | 32/67 (47.8%) | 32/67 (47.8%) | 1.000 |
| Chronic renal failure | 18/67 (26.9%) | 16/67 (23.9%) | 0.691 |
| Hyperlipidemia | 43/67 (64.2%) | 43/67 (64.2%) | 1.000 |
| **Laboratory data** |  |  |  |
| BNP level, pg/ml | 594.0 (342.0,1917.0) | 367.0 (133.0,1044.0) | 0.032 |
| Creatine level, umol/l | 80.6 (65.5,108.2) | 74.3 (60.6,115.2) | 0.488 |
| eGFR, mL/(min·1.73 m^2^) | 74.8±35.4 | 79.6±37.8 | 0.685 |
| Hemoglobin A1C, % | 7.0±1.9 | 6.9±1.5 | 0.644 |
| Triglyceride, mmol/l | 1.6 ±1.0 | 1.3±0.7 | 0.110 |
| LDL-c, mmol/l | 2.2±0.9 | 2.3±0.9 | 0.291 |
| HDL-c, mmol/l | 0.9±0.3 | 1.0±0.3 | 0.199 |
| Total cholesterol, mmol/l | 3.7±1.2 | 4.2±1.2 | 0.049 |
| Hs-CRP, mg/l | 6.3 (2.2,16.5) | 5.8 (2.5,13.6) | 0.487 |
| Troponin I, ng/ml | 0.09 (0.03,0.20) | 0.04 (0.01,0.13) | 0.135 |
| Elabela, ng/ml | 1.9 (1.6,2.3) | 5.4 (4.8,6.7) | <0.001 |
| **Echocardiographic data** |  |  |  |
| LAD, mm | 45.8±6.9 | 43.1±7.7 | 0.031 |
| LVEDd, mm | 57.9±8.9 | 52.5±7.9 | <0.001 |
| LVEDs, mm | 44.7±11.0 | 37.9±10.6 | <0.001 |
| PASP, mmHg | 30 (27,52) | 28 (23,44) | 0.003 |
| LVEF, % | 42.4 ±13.9 | 48.9±13.4 | 0.006 |
| **NYHA function class** |  |  |  |
| Class II | 16/67 (23.9%) | 28/67 (41.8%) | 0.047 |
| Class III | 29/67 (43.3%) | 15/67 (22.4%) | 0.010 |
| Class IV | 22/67 (32.8%) | 24/67 (35.8%) | 0.716 |
| **Medical therapy** |  |  |  |
| ACEI or ARB | 30/67 (44.8%) | 31/67 (46.3%) | 0.682 |
| beta blocker | 37/67 (55.2%) | 41/67 (61.2%) | 0.484 |
| Loop diuretic | 40/67 (59.7%) | 40/67 (59.7%) | 1.000 |
| MRA | 36/67 (53.7%) | 34/67 (50.7%) | 0.729 |
| Digoxin | 12/67 (17.9%) | 16/67 (23.9%) | 0.395 |
| Statins | 47/67 (70.1%) | 42/67 (62.7%) | 0.360 |
| **Endpoint** |  |  |  |
| Length of hospital stay day, days | 6.0 (4.0,10.0) | 6.0 (4.0,10.0) | 0.715 |
| Heart failure readmission | 15/67 (22.4%) | 5/67 (7.5%) | 0.015 |
| All-cause mortality | 4/67 (6.0%) | 3/67 (4.5%) | 0.698 |
| MACE | 19/67 (28.4%) | 8/67 (11.9%) | 0.018 |

BNP, brain natriuretic peptide; eGFR, estimated glomerular filtration rate; LDL-c, low density lipoprotein cholesterol; HDL-c, high density lipoprotein cholesterol; hs-CRP, high-sensitivity C-reactive protein; LAD, left atrial diameter; LVEDd, left ventricular end-diastolic dimension; LVEDs, left ventricular end-systolic diameter; PASP, pulmonary arterial pressure; LVEF, left ventricular ejection fraction; MRA, mineralocorticoid receptor antagonists. MACE, major adverse cardiac events.
