# Supplementary material for: Lower Plasma Elabela Levels in Hypertensive Patients With Heart Failure Predict the Occurrence of Major Adverse Cardiac Events: A Preliminary Study
Source: Front Cardiovasc Med. 2021 Mar 2;8:638468. doi: 10.3389/fcvm.2021.638468 (PMC7960768; doi:10.3389/fcvm.2021.638468)
Supplement: Supplementary file 3 [file Table_3.DOCX]

**Supplementary Table 3. The Baseline of Characteristics between Favorable and Unfavorable Outcome Group**

|  | Favorable outcome group  (n=107) | Unfavorable outcome group  (n=27) | P-value |
| --- | --- | --- | --- |
| Age, years | 68.1±11.1 | 71.7±10.9 | 0.138 |
| Male sex | 67/107(62.6%) | 19(70.4%) | 0.453 |
| BMI, kg/m^2^ | 25.4±3.4 | 24.9±3.6 | 0.460 |
| **Comorbidities** |  |  |  |
| Coronary heart disease | 79/107 (73.8%) | 15/27 (55.6%) | 0.064 |
| Atrial fibrillation | 46/107 (43.0%) | 11/27 (40.7%) | 0.833 |
| Diabetes Mellitus | 52/107 (48.6%) | 12/27 (44.4%) | 0.699 |
| Chronic renal failure | 24/107 (22.49%) | 10/27 (37.0%) | 0.119 |
| Hyperlipidemia | 72/107 (67.3%) | 14/27 (51.9%) | 0.135 |
| **Laboratory data** |  |  |  |
| BNP level, pg/ml | 373.0 (137.0,1157.0) | 1719.0 (501.0,3428.0) | <0.001 |
| Creatine level, umol/l | 73.3 (64.5,102.8) | 98.9 (71.6,153.0) | 0.006 |
| eGFR, mL/(min·1.73 m^2^) | 82.4±39.8 | 56.4±32.6 | 0.007 |
| Hemoglobin A1C, % | 7.0±1.7 | 6.8±1.5 | 0.538 |
| Triglyceride, mmol/l | 1.4 ±0.8 | 1.6±0.9 | 0.256 |
| LDL-c, mmol/l | 2.3±0.9 | 2.1±0.7 | 0.433 |
| HDL-c, mmol/l | 1.0±0.3 | 0.9±0.3 | 0.664 |
| Total cholesterol, mmol/l | 4.0±1.2 | 3.8±1.3 | 0.359 |
| hs-CRP, mg/L | 5.0 (2.2,13.8) | 10.7 (6.9,15.4) | 0.042 |
| Troponin I, ng/mL | 0.05 (0.01,0.20) | 0.09 (0.04,0.15) | 0.257 |
| Elabela, ng/mL | 4.1 (2.0,5.4) | 1.9 (1.6,5.1) | 0.003 |
| **Echocardiographic data** |  |  |  |
| LAD, mm | 44.0±7.5 | 46.0±6.9 | 0.213 |
| LVEDd, mm | 55.2±8.9 | 55.1±8.5 | 0.941 |
| LVEDs, mm | 41.1±11.4 | 42.3±10.9 | 0.629 |
| PASP, mmHg | 28 (25, 45) | 42 (28,54) | 0.013 |
| LVEF, % | 46.8±13.8 | 41.0±14.0 | 0.055 |
| **NYHA function class** |  |  |  |
| Class II | 43/107 (40.2%) | 1/27 (3.7%) | <0.001 |
| Class III | 34/107 (31.8%) | 10/27 (37.0%) | 0.603 |
| Class IV | 30/107 (28.0%) | 16/27 (59.3%) | 0.002 |
| **Medical therapy** |  |  |  |
| ACEI or ARB | 49/107 (45.8%) | 12/27 (44.4%) | 0.900 |
| beta blocker | 61/107 (57.0%) | 17/27 (63.0%) | 0.575 |
| Loop diuretic | 65/107 (60.7%) | 15/27 (55.6%) | 0.623 |
| MRA | 53/107 (49.5%) | 17/27 (63.0%) | 0.212 |
| Digoxin | 23/107 (21.5%) | 5/27 (18.5%) | 0.734 |
| Statins | 71/107 (66.4%) | 18/27 (66.7%) | 0.976 |

eGFR, estimated glomerular filtration rate; LDL-c, low density lipoprotein cholesterol; HDL-c, high density lipoprotein cholesterol; hs-CRP, high-sensitivity C-reactive protein; LAD, left atrial diameter; LVEDd, left ventricular end-diastolic dimension; LVEDs, left ventricular end-systolic diameter; PASP, pulmonary arterial pressure; LVEF, left ventricular ejection fraction; MRA, mineralocorticoid receptor antagonists.
