# Supplementary material for: Lower Plasma Elabela Levels in Hypertensive Patients With Heart Failure Predict the Occurrence of Major Adverse Cardiac Events: A Preliminary Study
Source: Front Cardiovasc Med. 2021 Mar 2;8:638468. doi: 10.3389/fcvm.2021.638468 (PMC7960768; doi:10.3389/fcvm.2021.638468)
Supplement: Supplementary file 4 [file Table_4.docx]

**Supplementary Table 4.** **Predictors of Baseline Characteristics for Major Adverse Cardiac Events in Univariate analysis and Multivariate Analysis in Hypertensive Patients with Heart Failure**

|  | Univariate analysis | | Multivariate analysis | |
| --- | --- | --- | --- | --- |
|  | HR (95% CI) | P value | HR (95% CI) | P value |
| Log_10_BNP | 5.05  (2.28-11.17) | <0.001 | 4.04  (1.82-9.00) | 0.001 |
| eGFR | 0.98  (0.97-0.99) | 0.006 |  |  |
| Elabela, ng/ml | 0.73  (0.58-0.91) | 0.006 | 0.75  (0.61-0.99) | 0.048 |
| Classification of NYHA | 3.16  (1.74-5.74) | <0.001 |  |  |
| PASP, mmHg | 1.03 |  |  |  |
|  | (1.00-1.05) | 0.025 |  |  |

BNP, brain natriuretic peptide; eGFR, estimated glomerular filtration rate; NYHA, New York Heart Association; PASP, pulmonary arterial pressure.
