# Supplementary figures and images for: Lower Plasma Elabela Levels in Hypertensive Patients With Heart Failure Predict the Occurrence of Major Adverse Cardiac Events: A Preliminary Study
Source: Front Cardiovasc Med. 2021 Mar 2;8:638468. doi: 10.3389/fcvm.2021.638468 (PMC7960768; doi:10.3389/fcvm.2021.638468)

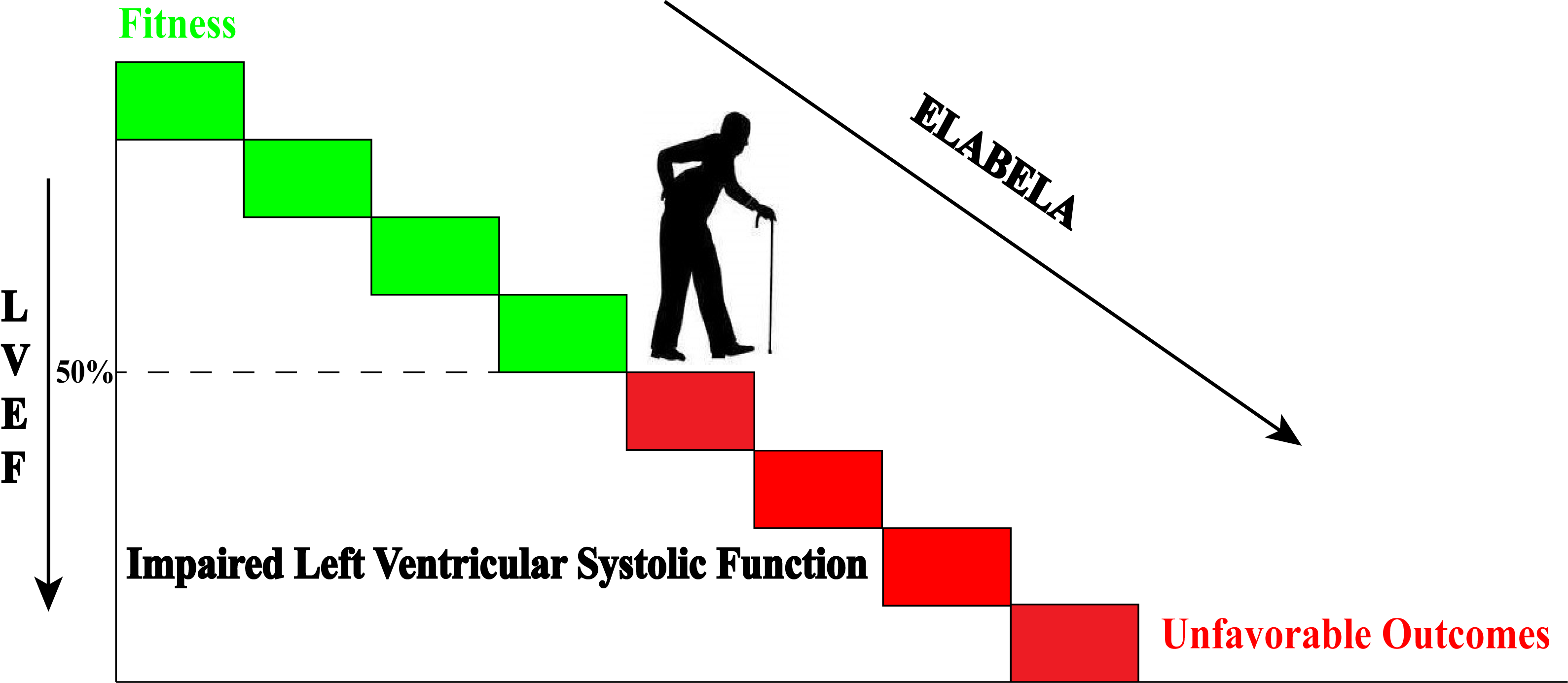

Supplement: Supplementary file 5 [file Image_5.TIF]

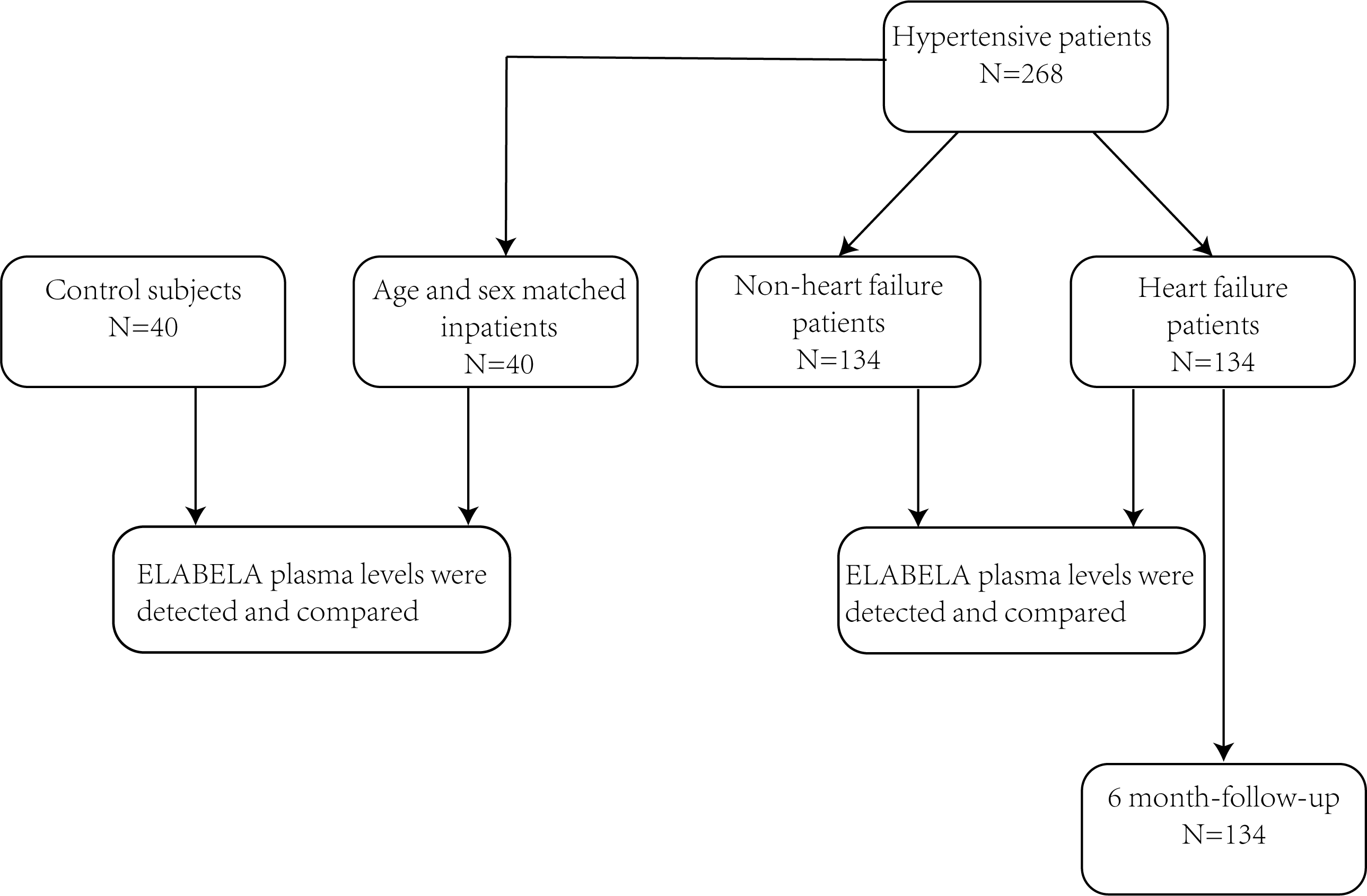

Supplement: Supplementary Figure 1 — Flow diagram for study from enrollment to follow-up. [file Image_1.TIF]

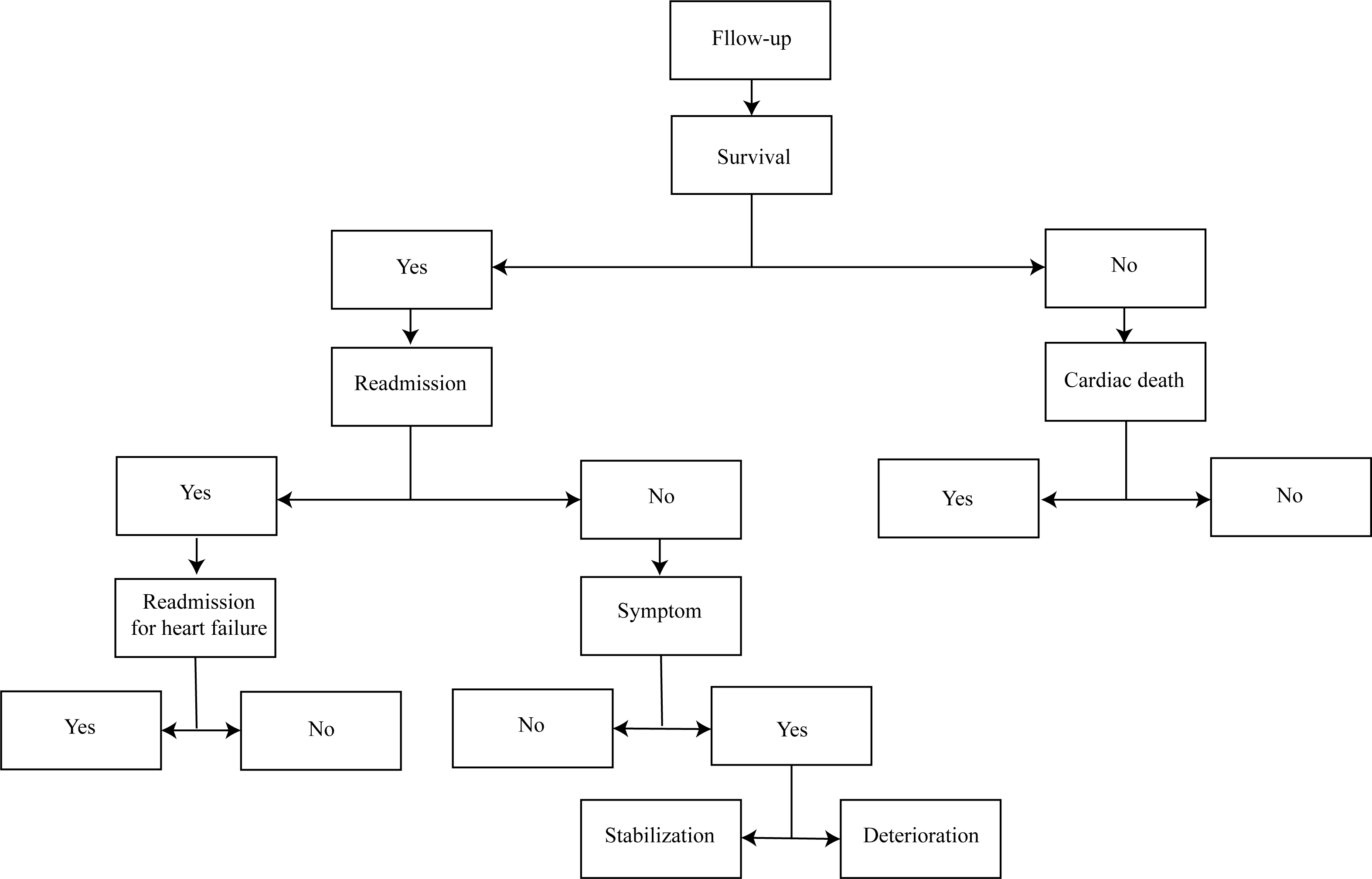

Supplement: Supplementary Figure 2 — Flow diagram for follow-up. Firstly, whether the subject is alive or not were confirmed. If the subject has died, the death details will be further investigated and death certificate will provide important information. Secondly, whether the subject is readmission or not were confirmed. If yes, we will further get the details information including the symptom, the diagnosis and medication records. Our team will clarify the reason of readmission. If no, we will ask the subject whether he/she had any symptoms associated with the deterioration of heart failure. If yes, we will assess the necessity of further outpatient visits and readmission. [file Image_2.TIF]

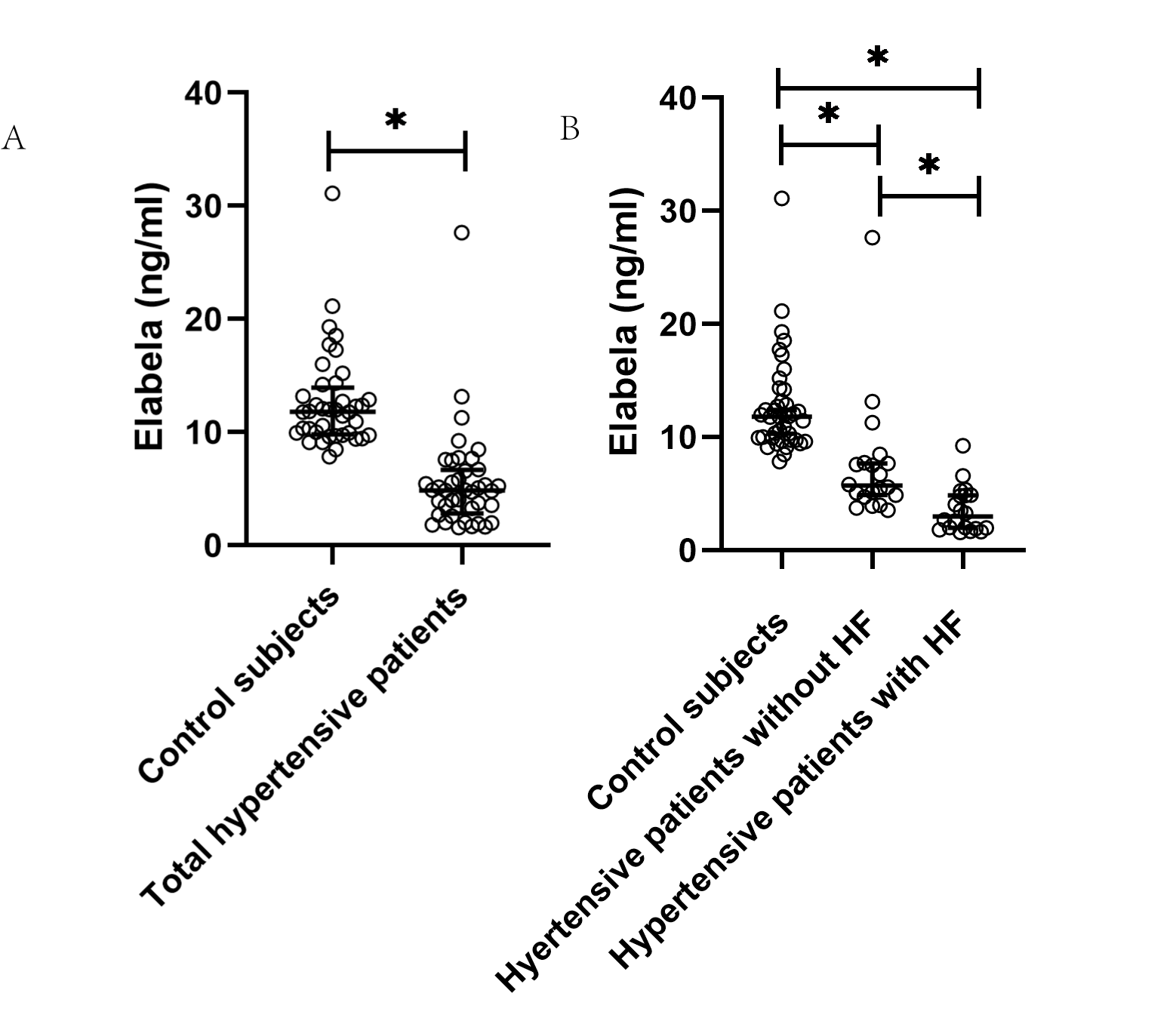

Supplement: Supplementary Figure 3 — Plasma Elabela levels in control subjects and age-sex-matched hypertensive patients. (A) The comparison between control subjects and hypertensive patients; (B) The comparison among control subjects, hypertensive patients with and without HF. [file Image_3.TIF]

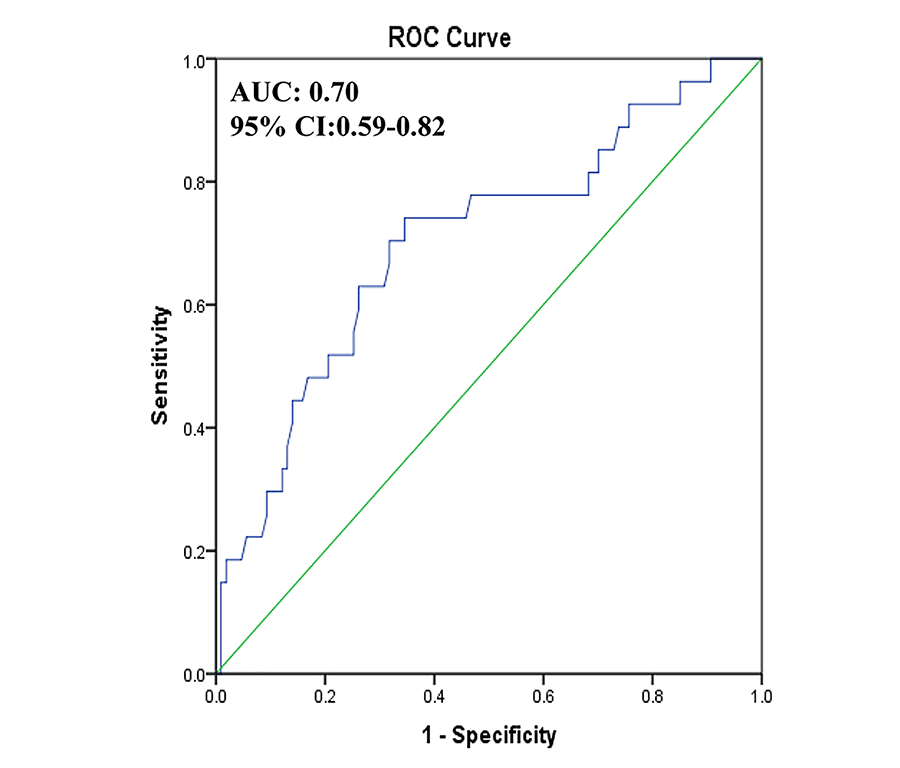

Supplement: Supplementary Figure 4 — ROC curve of the prognostic value of plasma Elabela levels in predicting major adverse cardiac events in patients with HF. [file Image_4.TIF]
